# Single-Suture versus Multiple-Suture Techniques Regarding Postoperative Pain, Trismus, Edema, Ecchymosis, and Operative Time in Surgical Removal of Impacted Mandibular Wisdom Teeth: A Clinical Trial 

**DOI:** 10.30476/dentjods.2024.102038.2332

**Published:** 2025-09-01

**Authors:** Mohammad Mehdizadeh, Abolfazl Karkoubzadeh

**Affiliations:** 1 Dept. of Oral and Maxillofacial Surgery, Faculty of Dentistry, Qom University of Medical Sciences and Health Services, Qom, Iran.; 2 Student Research Committee, Faculty of Dentistry, Qom University of Medical Sciences and Health Services, Qom, Iran.

**Keywords:** Tooth, Impacted, Mandible, Edema, Suture Techniques

## Abstract

**Background:**

Surgical removal of impacted mandibular wisdom teeth may be associated with postoperative complications such as infection, bleeding, edema, pain, ecchymosis and trismus. It seems that the number of sutures and the duration of surgery for impacted wisdom teeth are among the factors affecting the aforementioned complications.

**Purpose:**

This study aimed to compare single-suture versus multiple-suture techniques regarding postoperative pain, trismus, edema, ecchymosis, and operating time in surgical removal of impacted mandibular wisdom teeth by envelope flap surgery.

**Materials and Method:**

This double-blind split-mouth randomized controlled clinical trial was conducted on 30 patients requiring bilateral surgical extraction of impacted wisdom teeth with the same level of impaction through an envelope flap. In each patient, wisdom teeth of one randomly selected quadrant was extracted through an envelope flap and single-suture technique (experimental group) while the wisdom teeth of the other quadrant was extracted through an envelope flap by multiple-suture technique (control group). The two groups were compared regarding operative time, and also pain score, trismus (mouth opening), edema, and ecchymosis at 1, 3 and 7 days postoperatively using paired t-test (alpha= 0.05).

**Results:**

The two groups had no significant difference in pain score, edema, and ecchymosis at any time point (*p*> 0.05). The operative time (*p*= 0.005) was significantly longer, and mouth opening at 1, 3 and 7 days postoperatively (*p*< 0.05) was significantly smaller in the multiple-suture group.

**Conclusion:**

In the present study, postoperative trismus was significantly lower in the single-suture group than multiple-suture group, and the multiple-suture group had significantly longer operative time. Thus, single-suture technique appears to be superior to multiple-suture technique, and may be suggested for surgical removal of impacted mandibular wisdom teeth.

## Introduction

Impacted mandibular wisdom teeth surgery may be associated with postoperative complications such as infection, bleeding, edema, pain, and trismus [ 1 - 2 ]. Inflammation and bleeding following surgical extraction of impacted third molars can adversely affect the quality of life and daily activities of patients [ 3 ]. The prevalence of postoperative infection, edema, and inflammation is higher following impacted mandibular wisdom teeth surgery compared to maxillary wisdom teeth. Complex impacted wisdom teeth surgery, such as those with class III impaction or class C depth requires much more attention [ 1 ]. 

It has been reported that in surgical removal of impacted mandibular wisdom teeth, complete closure of surgical wound would result in postoperative edema and facial swelling, and would lead to discomfort and mouth opening limitation (trismus) [ 4 ]. A previous study also confirmed that complete wound closure is responsible for greater postoperative pain and edema in a significant number of patients [ 5 ]. 

Some strategies have been proposed to prevent or minimize postoperative complications. According to some researchers, techniques that allow the release of inflammatory exudates would decrease postoperative pain, edema, and trismus. Thus, further attention has been directed to such techniques [ 1 ]. 

Considering the adverse effects of postoperative pain, edema, trismus, and ecchymosis on patients’ quality of life, this study aimed to compare single-suture versus multiple-suture techniques regarding postoperative pain, trismus, edema, ecchymosis, and operative time in impacted mandibular third molars surgery by envelope flap. The null hypothesis of the study was that the single-suture and multiple-suture techniques would have no significant difference in the abovementioned parameters. 

## Materials and Method

This study was conducted at the Oral and Maxillofacial Surgery Department of School of Dentistry, Qom University of Medical Sciences between 2019 and 2020. The study protocol was approved by the Ethics Committee of the University (IR.MUQ.REC.1397.190) and registered in the Iranian Registry of Clinical Trials (Trial ID 42768). 

A double-blind split-mouth randomized controlled clinical trial was designed in which, the extraction site was sutured with the single-suture technique in the experimental side and with the multiple-suture technique in the control side. The results were reported in accordance with the Consolidated Standards of Reporting Trials. 

The inclusion criteria were patients requiring bilateral removal of impacted mandibular wisdom teeth with the same level of impaction according to the Pell and Gregory classification [ 6 ], as well as no systemic disease, no tobacco use, mental health, no infection or caries in mandibular third molars, and no infection of the adjacent teeth [ 7 ]. 

The exclusion criteria were unwillingness for participation in the study, local infection, pericoronitis, and complex impactions requiring elevation of a triangular flap. The sample consisted of 30 patients requiring bilateral extraction of impacted mandibular third molars who were selected among those presenting to a private dental office and dental clinic of School of Dentistry of Qom University of Medical Sciences by convenience sampling. 

Written informed consent was obtained from all patients for surgery of their impacted wisdom teeth and participation in the study. In each patient, one quadrant of the mandible was randomly assigned to the single-suture technique (intervention group) and the other quadrant was assigned to the multiple-suture technique (control group). 

The patients rinsed 0.2% chlorhexidine mouthwash (Irsha, Iran) before surgery, and prepping and draping were performed [ 7 ]. Inferior alveolar nerve block and long buccal anesthesia were administered by injection of 2% lidocaine plus 1:100,000 epinephrine [ 7 ]. After anesthesia induction, an envelope mucoperiosteal flap was elevated from the distobuccal of first molar to distal of third molar using a #15 surgical scalpel (Unicut, India). Bone was then removed by a round carbide bur and high-speed hand-piece under copious saline irrigation to access the tooth (BMT, Poland) [ 8 ]. The tooth was then sectioned by high-speed hand-piece, and removed by using an elevator . After irrigation of the extraction socket, the flap was returned, and the incision site was sutured using 3-0 silk sutures. In the intervention side, only one suture was applied distal to second molar tooth. In the control side, in addition to the abovementioned suture, another suture was applied at the interdental papilla between the first and second molars, and another one was applied distal to the third molar extraction socket (a total of three sutures).

Finally, a gauze pad was placed over the site of surgery, and the patients were instructed to retain it for 40 minutes. Patients also received postoperative instructions [ 7 ], which included putting an ice pack on the skin over the surgical side frequently for the first 24 hours after surgery [ 7 ], and intake of 500 mg acetaminophen 4 times a day for 3 days or another analgesic (in case of poor analgesic efficacy of acetaminophen). Type of prescribed analgesic was the same after both surgical procedures [ 7 ]. A minimum of 2-week interval was considered for complete resolution of pain, edema, and trismus between the two surgical procedures in the right and left quadrants. The patients had no pain or inflammation in the face prior to surgery [ 7 ]. Also, all surgical procedures for all patients were performed by the same surgeon in the same setting [ 7 ]. The following parameters were measured at 0, 1, 3, and 7 days after surgery [ 9 ]:

### Pain

A visual analog scale (VAS) was used for measurement of pain . 

### Edema

To assess postoperative edema, the distance between the lip corner and the lowest point of attachment of the ear lobe of the same side was measured horizontally by using a string and measuring its length by a ruler [ 7 ]. Also, the distance between the external eye canthus and the angle of mandible was measured vertically at the respective side [ 1 ]. 

### Ecchymosis

Facial color change and bruising at the site (indicative of ecchymosis) were recorded. The surface area of the discolored region due to ecchymosis was also calculated by photographing the site and calculating the surface area using Digimizer v4.1.1.0 software. Type of discoloration (brownish, greenish, yellowish, and reddish) was also recorded. 

### Trismus

Mouth opening was measured as the distance between the mesio-incisal angle of maxillary right central incisor and the same point in the mandibular right central incisor [ 10 ].

### Operative time

Operative time was recorded as the time lapse between the initial incision and final mucosal closure (applying the last suture).

### Sample size calculation

The minimum sample size was calculated to be 26 patients according to a previous study [ 11 ], assuming study power of 95%, type 1 error of 1%, and minimum mean difference in pain score to be 1.9 at 1 and 7 days, postoperatively, and standard deviation of pain score to be 0.61 and 0.92 in the two groups using MedCal software. Considering the interventional design of the study and the possibility of loss to follow-up to be 15% in the intervention group, the sample size was increased to 30 patients. 

No interim analyses were performed and no stopping guidelines were established. Allocation of suturing technique to the quadrants was performed randomly by flipping a coin. The patients were blinded to the group allocation of their quadrants. Also, the examiner who examined and recorded the parameters was blinded to the group allocation of quadrants.

### Statistical analysis

Data were analyzed using SPSS version 25 (SPSS Inc., IL, USA) by paired t-test at 0.05 level of significance. 

## Results

### Participant flow

The sample consisted of 30 patients including 18 females (60%) and 12 males (40%) with a mean age of 23.23 years (range 18 to 34 years). The intervention group (single-suture technique) included 18 left-side (60%) and 12 right-side (40%) impacted mandibular third molars. The control group (three-suture technique) included 18 right-side (60%) and 12 left-side (40%) impacted mandibular third molars, which were randomly selected. 

### Harms

No patients were harmed during the study.

### Primary outcomes

Ecchymosis

In the intervention group (one-suture), no evidence of ecchymosis was seen on days 1, 3 and 7. In the control group (three-suture), no evidence of ecchymosis was seen on day 1. On day 3, one patient (3.3%) had ecchymosis, which resolved by day 7. 

### Pain

The minimum and maximum pain score in the intervention group was 0 and 8 (out of 10) on day 1. The maximum pain score in the intervention group decreased to 6 and 5 in 3 and 7 days (Table 1). The minimum and maximum pain score in the control group was 0 and 9 (out of 10) on day 1. The maximum pain scores decreased on days 3 and 7 to 8 and 5 (Table 2). The difference in pain was not significant between the two groups at any time point (*p*> 0.05, Table 3).

**Table 1:** Measures of central dispersion for pain, edema, mouth opening, and operative time in the intervention group

Variable	Minimum	Maximum	Mean±std. deviation
Pain on day 1	0	8	43.3±66.2
Pain on day 3	0	6	33.2±10.2
Pain on day 7	0	5	90.±37.1
Vertical edema on day 0	1.9	3.12	570.10±72.0
Horizontal edema on day 0	3.8	3.12	837.9±82.0
Vertical edema on day 1	6.9	2.13	953.10±79.0
Horizontal edema on day 1	9.8	3.12	417.10 ±96.0
Vertical edema on day 3	6.9	9.12	787.10±74.0
Horizontal edema on day 3	1.9	0.12	277.10±75.0
Vertical edema on day 7	0.9	0.13	537.10±74.0
Horizontal edema on day 7	6.8	1.12	900.9±70.0
Mouth opening (mm) on day 0	7.3	2.6	733.4±65.0
Mouth opening (mm) on day 1	7.1	5.5	480.3±03.1
Mouth opening (mm) on day 3	1.2	5.5	947.3±94.0
Mouth opening (mm) on day 7	4.3	8.5	497.4±70.0
Operative time	40.4	00.21	2477.8 ±12.3

**Table 2:** Measures of central dispersion for pain, edema, mouth opening, and operative time in the control group

Variable	Minimum	Maximum	Mean±std. deviation
Pain on day 1	0	0.9	70.3±2.66
Pain on day 3	0	8	60.2±2.17
Pain on day 7	0	5	97.±1.27
Vertical edema on day 0	2.9	1.12	733.10±0.79
Horizontal edema on day 0	7.7	5.12	777.9±0.79
Vertical edema on day 1	4.9	3.13	010.11±0.95
Horizontal edema on day 1	5.8	1.13	523.10±0.99
Vertical edema on day 3	5.9	4.12	677.10±0.68
Horizontal edema on day 3	6.8	5.12	257.10±0.90
Vertical edema on day 7	2.9	0.13	510.10±0.90
Horizontal edema on day 7	3.8	3.12	993.9±0.83
Mouth opening (mm) on day 0	7.3	0.6	474.4±0.60
Mouth opening (mm) on day 1	5.1	1.5	983.3±0.94
Mouth opening (mm) on day 3	3.2	3.5	503.3±0.77
Mouth opening (mm) on day 7	9.2	9.5	230.4±0.73
Operative time	31.5	46.23	7513.10±3.87

**Table 3:** Comparison of pain, edema, mouth opening, and operative time between the two groups by paired t-test

Variable	Intervention Mean±std. deviation	Control Mean±std. deviation	*p* Value
Pain on day 1	43.3±66.2	7.3±66.2	631.
Pain on day 3	33.2±10.2	60.2±7.2	482.
Pain on day 7	099.±37.1	097.±27.1	831.
Vertical edema on day of surgery	57.10±72.0	73.10±79.0	097.
Horizontal edema on day of surgery	83.9±82.0	77.9±79.0	481.
Vertical edema on day 1	95.10±79.0	01.11±95.0	654.
Horizontal edema on day 1	41.10±96.0	52.10±99.0	583.
Vertical edema on day 3	78.10±74.0	67.10±68.0	210.
Horizontal edema on day 3	27.10±75.0	25.10±90.0	890.
Vertical edema on day 7	53.10±74.0	51.10±90.0	792.
Horizontal edema on day 7	90.9±70.0	99.9±83.0	449.
Mouth opening on day 0	73.4±65.0	74.4±60.0	838.
Mouth opening on day 1	48.3±03.1	98.2±94.0	007.
Mouth opening on day 3	94.3±94.0	50.3±77.0	007.
Mouth opening on day 7	49.3±70.0	23.4±73.0	002.
Operative time	24.8±12.3	75.10±87.3	005.

### Mouth opening

The minimum and maximum mouth opening in the intervention group was 37mm and 60mm, respectively, which increased on 3 and 7 days (Table 1). The minimum and maximum mouth opening in the control group was 17mm and 55mm, respectively, which increased on days 3 and 7 (Table 2). The difference in mouth opening was significant between the two groups at all time points (*p*< 0.05, Table 3); such that mouth opening was significantly greater in the intervention group at all time points.

### Edema

The results regarding vertical and horizontal edema are shown in Tables 1 and 2. Edema was not significantly different between the two groups at any time point (*p*> 0.05, Table 3).

### Operative time

The minimum operative time in the intervention group was 4.40 minutes and the maximum operative time was 21 minutes (Table 1). In the control group, the minimum and maximum operative time was 5.31 minutes and 23.46 minutes, respectively (Table 2). The operative time was significantly shorter in the intervention group than the control group (Table 3, *p*= 0.005). 

### Secondary outcomes

### Laterality

Table 4 compares pain, trismus, edema, and operative time in the right and left sides. As shown, no significant difference was found in any parameter between the right and left sides (*p*> 0.05).

**Table 4:** Comparison of pain, trismus, edema, and operative time in the right and left sides by paired t-test

Variable	Right Mean±std. deviation	Left Mean±std. deviation	*p* Value
Pain on day 1	62.3±64.2	5.3±68.2	847.
Pain on day 3	70.2±246.2	23.2±012.2	400.
Pain on day 7	17.1±341.1	26.1±70.0	171.
Vertical edema on day of surgery	66.10±86.0	64.10±64.0	906.
Horizontal edema on day of surgery	83.9±77.0	78.9±84.0	800.
Vertical edema on day 1	86.10±94.0	097.11±97.0	310.
Horizontal edema on day 1	30.10±89.0	63.10±03.1	195.
Vertical edema on day 3	75.10±72.0	71.10±70.0	843.
Horizontal edema on day 3	24.10±82.0	29.10±83.0	805.
Vertical edema on day 7	30.10±85.0	57.10±79.0	828.
Horizontal edema on day 7	97.9±78.0	92.9±76.0	816.
Mouth opening on day 0	74.4±57.0	73.4±67.0	967.
Mouth opening on day 1	25.3± 05 .1	20.3±98.0	850.
Mouth opening on day 3	72.3±92.0	72.3±85.0	989.
Mouth opening on day 7	32.4±70.0	40.4±75.0	648.
Operative time	80.9±83.3	19.9±62.3	529.

### Gender

Table 5 compares pain, trismus, edema, and operative time in male and female patients. As shown, no significant diversity was found in any parameter between males and females (*p*> 0.05).

**Table 5:** Comparison of pain, trismus, edema, and operative time in males and females by paired t-test

Variable	Male Mean±std. deviation	Female Mean±std. deviation	*p* Value
Pain on day 1	58.2±46.2	4±70.2	712.
Pain on day 3	08.2±10.2	50.2±14.2	609.
Pain on day 7	83.0±40.1	94.0 ± 39.1	405.
Vertical edema on day of surgery	10.11±62.0	21.10±56.0	180.
Horizontal edema on day of surgery	24.10±89.0	56.9±66.0	258.
Vertical edema on day 1	23.11±94.0	76.10±64.0	272.
Horizontal edema on day 1	46.10±97.0	38.10±98.0	282.
Vertical edema on day 3	07.11±80.0	59.10±64.0	233.
Horizontal edema on day 3	35.10±86.0	22.10±68.0	250.
Vertical edema on day 7	86.10±85.0	31.10±58.0	245.
Horizontal edema on day 7	20.10±81.0	69.9±55.0	234.
Mouth opening on day 0	88.4±73.0	63.4±59.0	212.
Mouth opening on day 1	65.3±79.0	36.3±17.1	229.
Mouth opening on day 3	20.4±81.0	77.3±00.1	234.
Mouth opening on day 7	68.4±71.0	37.4±68.0	206.
Operative time	57.7±78.1	69.8±75.3	515.

## Discussion

The results showed no significant difference in pain and edema between the two groups at any time point. However, maximum mouth opening was significantly greater in the single-suture group at 1, 3 and 7 days, compared with the multiple-suture group. Operative time was also significantly shorter in the single-suture group. Thus, the null hypothesis of the study was partially accepted and partially rejected. 

Pasqualini *et al*. [ 12 ] reported that postoperative pain and edema in the single-suture group were lower than those in the multiple-suture closure, which was different from the present findings. This difference in the results may be due to the fact that the study by Pasqualini *et al*. [ 12 ] did not have a split-mouth design. Waite and Cherala [ 13 ] in their review study reported lower pain score and shorter operative time in single-suture technique. Their results regarding operative time were in agreement with the present findings; however, the difference in pain score was not significant between the two groups in the present study. This difference may be due to the fact that not all patients in their study were systemically healthy (there were 117 ASA I and 2 ASA II patients in their study). Also, smoking and pregnancy were the only exclusion criteria in their study, while it is apparent that people with underlying diseases may use medications that can alter the research results. For instance, the use of steroidal and non-steroidal anti-inflammatory drugs in cardiopulmonary patients or autoimmune diseases may change the outcome. 

Danda *et al*. [ 8 ] reported lower pain and edema in the single-suture group, which may be due to postoperative prescription of amoxicillin and diclofenac for patients in their study while antibiotics were not prescribed for patients in the present study. Maria *et al*. [ 11 ] also showed lower level of pain, edema, and trismus in the single-suture technique. Their results regarding trismus were in line with the present findings, while their results regarding pain and edema were different. Osunde *et al*. [ 10 ] demonstrated significantly lower trismus, pain, and edema in the single-suture group only in the first 3 days while in the present study, trismus was significantly lower at all time points in the single-suture group.

The difference between their results and the present findings may be due to split-mouth design of the present study. It should be noted that in split-mouth studies, interfering factors such as systemic diseases or psychological differences caused by the inherent differences of different people are eliminated.

 Hashemi *et al*. [ 7 ] compared three-suture technique with no suturing and reported significantly lower pain and edema in no suturing side. They also prescribed antibiotics; thus, their results cannot be precisely compared with the present findings. In another study, Osunde *et al*. [ 1 ] reported lower level of pain, edema, and trismus on 1 and 3 days postoperatively in the single- suture technique; however, the difference at 7 days was not significant in any parameter. They also reported significantly shorter operative time in the single-suture group, which was consistent with the present findings.

Koyuncu *et al*. [ 14 ] reported significantly lower pain, edema, trismus and operative time in the single-suture technique. Difference between their results and the present findings in pain and edema may be attributed to prescription of antibiotics in their study. Chaudhary *et al*. [ 15 ] found significantly lower pain score and edema in the single-suture group only on day 1. At 3 days, only the difference in edema was significant. Different surgical technique and smaller sample size in their study may explain the diversity between their results and the present findings. Pachipulusu [ 16 ] and Singh *et al*. [ 17 ] reported lower pain, edema, and trismus in the single-suture group, which was different from the present findings probably due to the fact that their study did not have a split-mouth design and as it was mentioned before, maximum compliance has not been observed in their studies.

Khaitan *et al*. [ 9 ] found no significant difference in pain score between the two groups while trismus was significantly lower in the single-suture group at all follow-up times. Their findings in this regard were in agreement with the present results. However, they also reported significantly lower edema on days 1 and 3 in the single-suture group. Takadoum *et al*. [ 18 ] found no significant difference in pain, edema, and trismus between the two groups. Their results regarding pain and edema were in line with the present findings. However, they obtained different results regarding trismus, which may be due to the fact that they evaluated smoker patients. Unlike the present study, Aggarwal *et al*. [ 19 ] reported significantly lower pain, edema, and trismus in the single-suture group. Their results regarding trismus were in accordance with the present results. 

In all the aforementioned studies, the operative time was longer in the multiple-suture group. Another noteworthy issue is the time spent on suture removal which would be obviously longer for removal of multiple sutures than removal of only one suture. However, this parameter was not evaluated in the present study, or previous investigations. Gender and laterality had no significant effect on the results in the present study. 

Split-mouth design was a major strength of the present study, which eliminated the effect of inter-individual differences as confounders on the results. Also, no antibiotic prophylaxis was performed to eliminate its possible confounding effect on postoperative pain and edema. Furthermore, ecchymosis was also compared between the two groups, which has not been evaluated in relevant previous studies. A 14-day time interval was considered between the two surgical procedures and the same type of analgesics were prescribed after both procedures, which are among other strengths of the present study. Future studies with a larger sample size are required to verify the present findings. Also, time required for suture removal should also be compared between the two groups in further studies. 

The most important limitation of the current research was the spread of the COVID-19 pandemic shortly after the start of the study, which caused some patients to withdraw themselves from the study and not willing to perform elective surgeries and consequently altering the statistical population.

## Conclusion

In the present study, postoperative trismus was significantly lower in the single-suture group than the multiple-suture group, and the multiple-suture group had significantly longer operative time. Thus, single-suture technique appears to be superior to multiple-suture technique, and may be suggested for surgical extraction of mandibular third molars.
